# Identification of mesenchymal stromal cell survival responses to antimicrobial silver ion concentrations released from orthopaedic implants

**DOI:** 10.1038/s41598-020-76087-1

**Published:** 2020-11-03

**Authors:** Paul Souter, John Vaughan, Kerry Butcher, Adam Dowle, Jim Cunningham, James Dodd, Michael Hall, Darren Wilson, Alan Horner, Paul Genever

**Affiliations:** 1 Smith and Nephew plc, 101 Hessle Road, Hull, HU3 4DJ UK; 2 Department of Biology, University of York, Wentworth Way, York, YO10 5DD UK

**Keywords:** Mesenchymal stem cells, Reverse transcription polymerase chain reaction, Proteomic analysis, Bone, Self-renewal, Stem-cell differentiation

## Abstract

Antimicrobial silver (Ag^+^) coatings on orthopaedic implants may reduce infection rates, but should not be to the detriment of regenerative cell populations, primarily mesenchymal stem/stromal cells (MSCs). We determined intramedullary silver release profiles in vivo, which were used to test relevant Ag^+^ concentrations on MSC function in vitro. We measured a rapid elution of Ag^+^ from intramedullary pins in a rat femoral implantation model, delivering a maximum potential concentration of 7.8 µM, which was below toxic levels determined for MSCs in vitro (EC_50_, 33 µM). Additionally, we present in vitro data of the reduced colonisation of implants by *Staphylococcus aureus*. MSCs exposed to Ag^+^ prior to/during osteogenic differentiation were not statistically affected. Notably, at clonal density, the colony-forming capacity of MSCs was significantly reduced in the presence of 10 µM Ag^+^, suggesting that a subpopulation of clonal MSCs was sensitive to Ag^+^ exposure. At a molecular level, surviving colony-forming MSCs treated with Ag^+^ demonstrated a significant upregulation of components of the peroxiredoxin/thioredoxin pathway and processes involved in glutathione metabolism compared to untreated controls. Inhibition of glutathione synthesis using l-buthionine sulfoxamine eliminated MSC clonogenicity in the presence of Ag^+^, which was rescued by exogenous glutathione.

## Introduction

Surgical site infection following orthopaedic trauma imparts a significant clinical and economic burden on patients and healthcare systems. It is estimated to double the median healthcare costs and patient hospitalisation time at centres within the United States^1^. In a European study specifically investigating infection of tibial fractures, treatment costs were estimated to increase by 6.5-times compared to patients with no complications^2^.

Persistent infections are primarily caused by the presence of bacterial biofilms attached to the surfaces of implanted materials, with colonisation of fracture fixation devices caused by *Staphylococcus aureus* in approximately 30% of cases^3^. These micro-colonies are highly organised, sessile communities surrounded by extracellular polymeric substances that are able to evade both the host’s defence mechanisms and antibiotics^4–9^. In vivo, the formation of a biofilm is greatly enhanced by the implantation of an inert material, and was the cause of failure observed in many patients^10,11^. It is therefore evident that the prevention of bacterial adhesion and subsequent biofilm formation can prevent implant-associated infection. With this in mind, gentamicin-coated intramedullary (IM) nails for use in fracture repair have been commercialised to block implant associated infection by Gram-negative bacteria^12^.

However, with the rise in antibiotic resistance, the use of a broad spectrum antimicrobial may be more appropriate. As a result, there has been growing interest in the use of silver for orthopaedic applications. In addition to the direct action on many microbial strains, the use of silver has also been shown to potentiate the effectiveness and broaden the bacterial species activity of antibiotics^13–15^. The wide bactericidal effects of silver are caused by disruption of the cell membrane, damage to DNA and prevention of replication, denaturation of proteins and enzymes that contribute to the loss of cell integrity as well as the disruption of energy metabolism, in part caused by the generation of reactive oxygen species (ROS)^16–20^. Currently, the application of silver to orthopaedic megaprostheses implanted during bone resection as a result of osteosarcoma has proven to reduce the incidence of infection in these compromised patients^21–23^. The application of silver to orthopaedic trauma devices such as intramedullary nails, could therefore have a similar clinical impact.

Fracture repair requires the action of immune cells, chondrocytes and osteoblasts, the numbers and regulation of which are influenced by mesenchymal stem/stromal cells (MSCs) that act as a source or progenitors, giving rise to reparative osteochondral tissue^24^. MSCs are supplied to the fracture site from the surrounding soft tissue, periosteum and bone marrow, all or which may be disrupted in a severe fracture, therefore preservation of existing MSC populations and their function is of importance^25^. Specifically, the impact of silver ions (Ag^+^) on repair of the fracture site and the subsequent generation of ROS that also occurs in eukaryotic cells, needs to be considered, particularly since the level of ROS can affect the lineage divergence of MSCs, with decreased osteogenesis occurring under in vitro conditions of oxidative stress^26–28^.

Here, we determined the Ag^+^ release profile from intramedullary implants to identify how exposure to relevant in vivo concentrations of silver ions affected MSC function and survival responses in vitro. Our findings provide insight into the potential clinical use of this metal for biofilm prevention in orthopaedic trauma situations.

## Results

### Synthesis and characterisation of intramedullary pin implants

Silver ions were integrated on to implant surfaces and inductively coupled plasma mass spectrometry (ICP-MS) quantification of total Ag^+^ confirmed nominal target loading of approximately 50 µg/cm^2^ (pins: mean, 50.36 µg/cm^2^ SEM ± 0.76, n = 6).

### *Staphylococcus aureus* adhesion to silver coated Ti64 grit blasted pins

**Figure 1:**
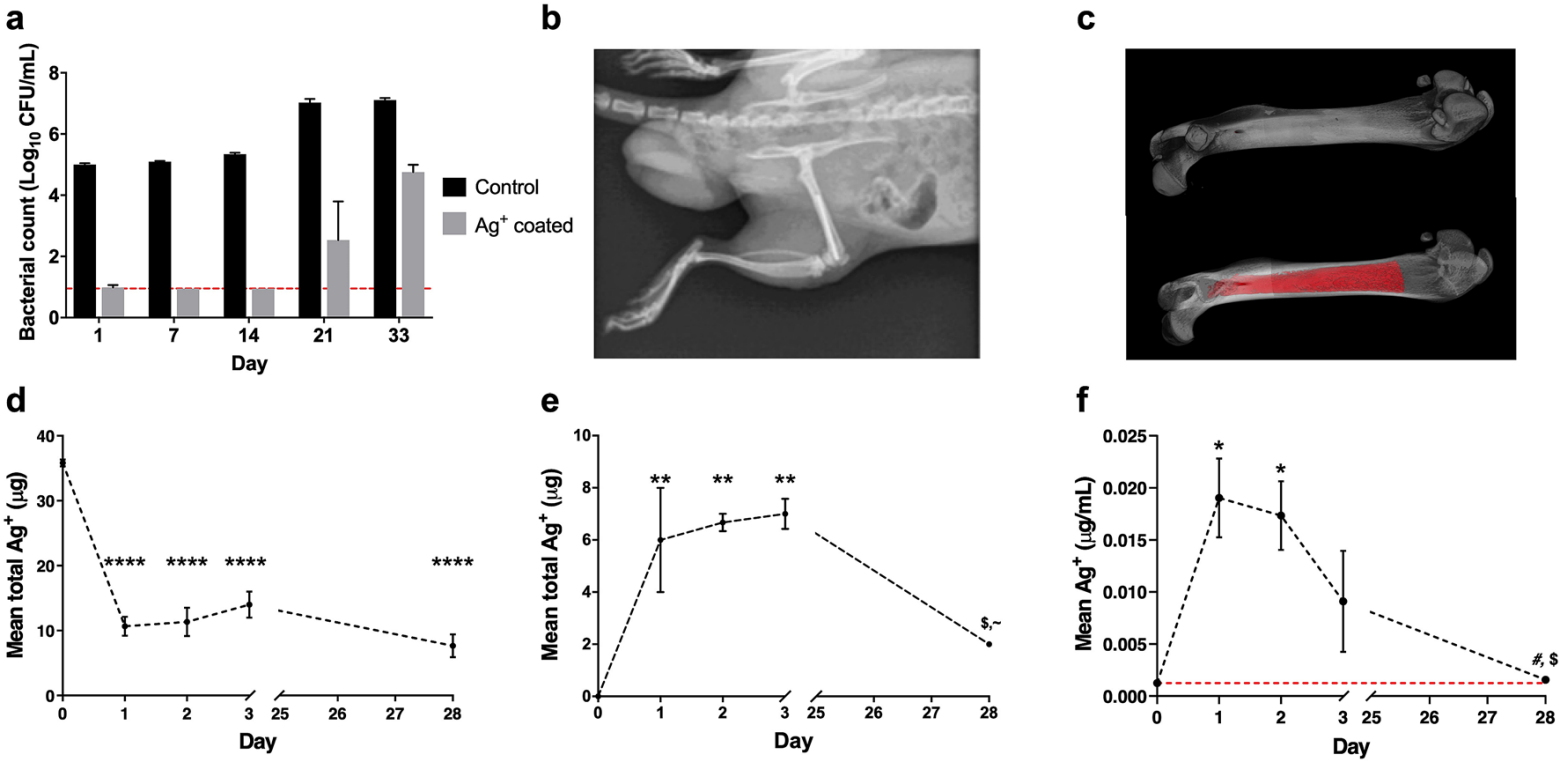
(**a**–**f**) Surface recovery of *S. aureus* from silver-coated grit blasted Ti64 pins following in vitro challenge (**a**), line represents the limit of detection of 0.95 CFU/mL, (**b**) confirmation of pin implantation during in vivo assessment of silver elution, (**c**) microCT scan of rat femur highlighting intramedullary canal volume. Measurement of total Ag from (**d**) recovered implants (µg), (**e**) operated femurs (µg), and silver concentration in (**f**) plasma (μg/mL), red line represents limit of detection (= 0.00125 μg/mL). All values are means ± SEM (n = 3) determined via ICP-MS for samples taken at termination (Day 1, 2, 3 and 28). Statistical analysis by one-way ANOVA using Tukey correction for multiple comparisons, significance indicated against Day 0 by *p < 0.05, **p < 0.01, ****p < 0.0001, Day 1 by ^#^*p* < 0.05, Day 2 by ^$^*p* < 0.05 and Day 3 by ^~^*p* < 0.05.

Control pin implants, without Ag^+^ coatings, supported bacterial colonisation over the test period, with a mean surface count of 5.00 log_10_ CFU/sample after 1 day rising to 7.11 log_10_ CFU/sample at day 33 (Fig. 1a). Colonisation was significantly reduced on Ag^+^ coated pins (> 4 log_10_ CFU/sample) over control counts at 24 h, maintaining this distinction at day 7 and 14 re-challenges (> 4 log_10_ CFU/sample). At these time points all replicates displayed counts at or below the detection limit. At day 21, Ag^+^ coated samples displayed a degree of colonisation, however values remained below control counts by > 4 log_10_ CFU/sample. Colonisation was detected in Ag^+^ coated pins at day 33 that was comparable to control samples at earlier time points (day 1, 7 and 14), but still reduced compared to control (Fig. 1a).

### Rapid elution of Ag^+^ from implants within intramedullary canal environment

To determine Ag^+^ elution kinetics, Ag^+^ coated titanium pins (Ag^+^, 50.36 µg/cm^2^ SEM ± 0.76) were implanted in to rat femoral intramedullary canals (Fig. 1b,c) and the Ag^+^ determined in the recovered implant (Fig. 1d), the femurs receiving the implants (Fig. 1e) and plasma (Fig. 1f). Results revealed a burst release within the 24 h following implantation, equating to approximately 70% (mean 25.15 μg, SEM ± 1.45 μg) of the pre-implantation content, with little significant release over the remaining study period (Fig. 1d). A corresponding rapid elevation in plasma and femoral Ag^+^ content was detected at Day 1 that remained elevated at Day 3 (Fig. 1e,f). Maximum plasma concentration (0.019 μg/mL, SEM ± 0.004 μg/mL) declined following the Day 1 peak and was reduced to baseline over the remaining study period. Femoral Ag^+^ content at Day 3 (7.00 μg; SEM ± 0.58 μg) significantly decreased by Day 28 (2 μg) (Fig. 1f).

The free canal volume (i.e. available canal volume with implant in situ) determined from microCT reconstructions (Fig. 1c) equated to 43 µL, therefore the theoretical maximum Ag^+^ concentration within the canal following initial release equated to 6.00 μM (mean 5.42 μM, SEM ± 0.31, Supplementary Table S1). In the event of instant total release of Ag^+^ from an implant, Ag^+^ within the canal was calculated to reach a peak of 7.97 μM (mean 7.72 μM, SEM ± 0.12). For the purpose of in vitro testing, an Ag^+^ value of 10 μM was taken forwards as a maximum test concentration. Further data detailing Day 1 implant elution, total plasma Ag^+^ and unaccounted Ag^+^ are available in Supplementary Table S1.

### In vitro susceptibility of MSCs to Ag^+^ is increased at clonal density

**Figure 2:**
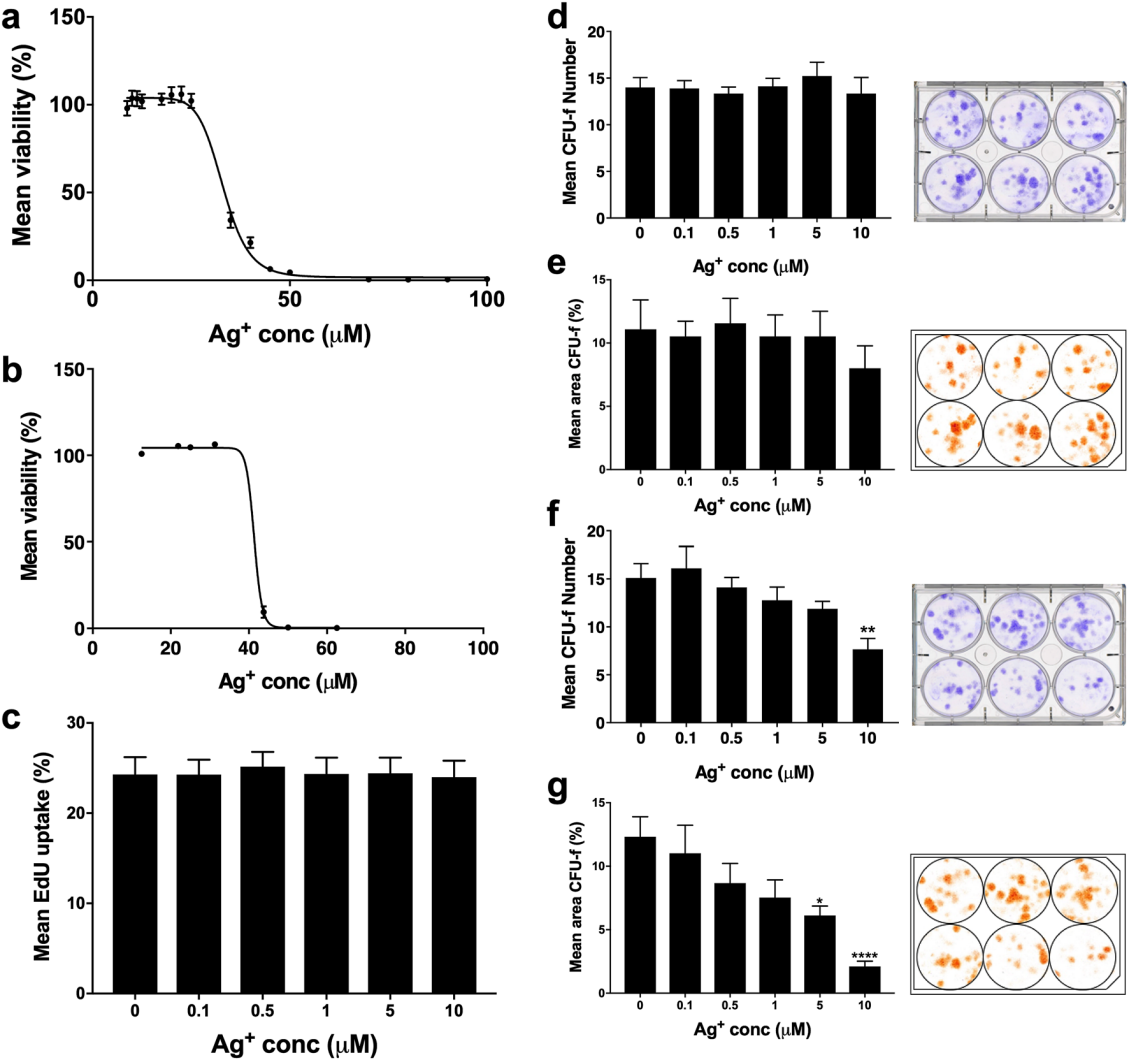
(**a**–**g**) Effect of Ag^+^ dose response on percentage viability of (**a**) hObs (EC_50_: 41.4 μM) and (**b**) MSCs (EC_50_: 33.06 μM). (**c**) proliferation (EdU) and CFU-f number (**d**) and area (**e**) of MSCs at sub-EC_50_ concentrations (≤ 10 µM) during 3 day exposure. CFU-f number (**f**) and area (**g**) during continuous Ag^+^ exposure. All values are means ± SEM (n = 3). Images in the right panel are representative of 6-well plates, stained with crystal violet (blue) and analysed (brown) for CFU-f number and area; Ag^+^ concentrations for the plates are (L–R): top row, 0, 0.1, 0.5 μM; bottom row, 1, 5, 10 μM. Statistical analysis was performed for all Ag^+^ groups compared to control (by one-way ANOVA using Dunnett’s correction for multiple comparisons), significance indicated by *p < 0.05, **p < 0.01, ****p < 0.0001.

The effect of Ag^+^ dose on primary MSC and human osteoblast (hObs) cultures provided calculated EC_50_ values of 33.06 μM and 41.4 μM, respectively (Fig. 2a,b). MSC proliferation in the presence of Ag^+^ was unaffected over 3 days at concentrations below the EC_50_ as determined by EdU incorporation (Fig. 2c). Colony-forming capacity of MSCs as measured by colony number and percentage area was also unaffected by exposure to sub-EC_50_ Ag^+^ concentrations during the initial 3 days of culture (which broadly replicated the exposure period determined during the in vivo elution study) (Fig. 2d,e). However, when MSCs were treated with Ag^+^ for the duration of the assay period, 10 µM Ag^+^ caused a significant reduction in colony number and area (Fig. 2f,g).

### MSC colony formation is unaffected by previous exposure to Ag^+^

**Figure 3:**
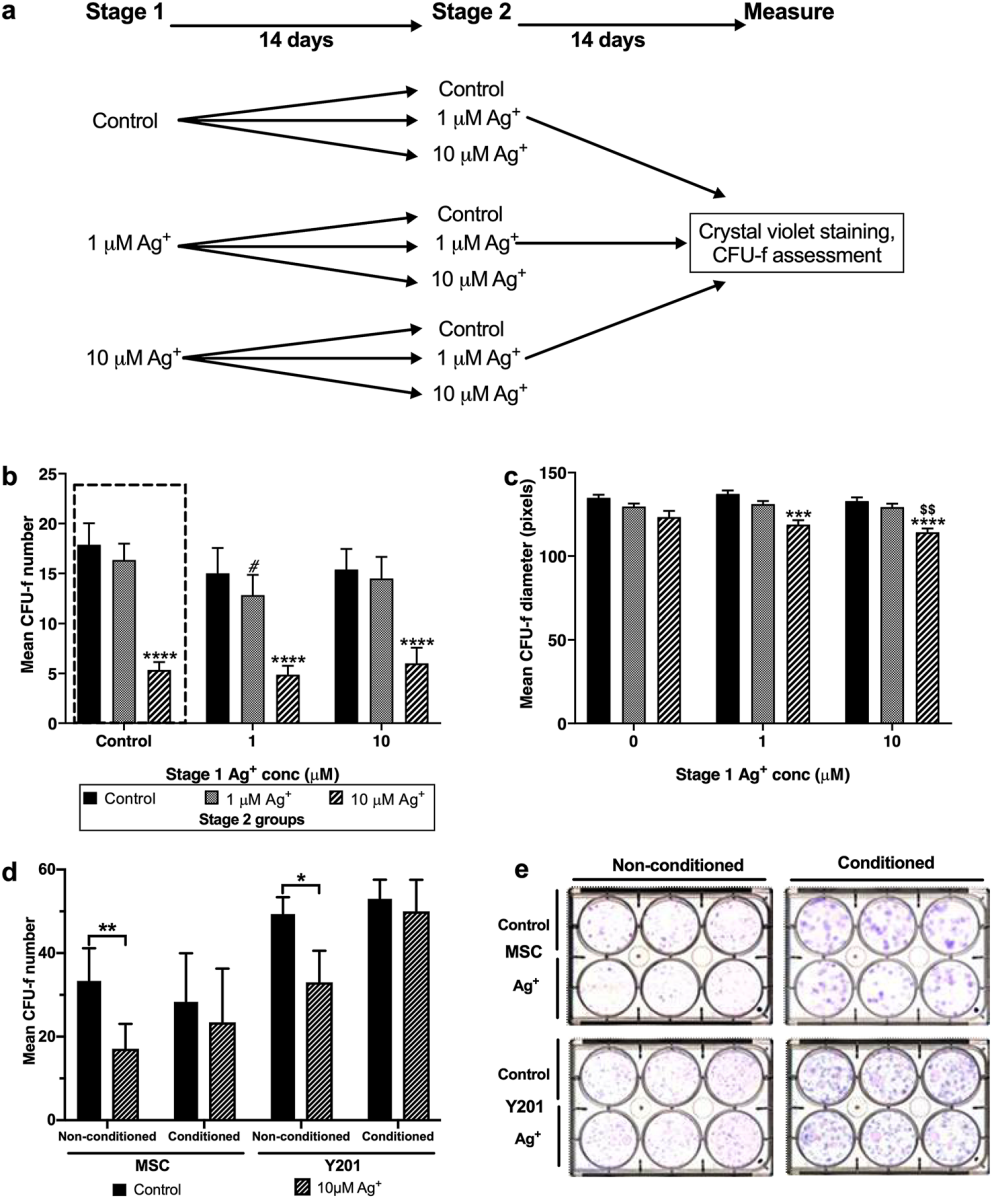
(**a**–**e**) Two-stage assay design (**a**) used to determine the continued MSC colony-forming capacity and Ag^+^ tolerance. Clonal density MSCs were cultured for 14 days ± Ag^+^ after which colonies of treatment groups were pooled and reseeded at clonal density and re-cultured ± Ag^+^ for a further 14 days. The resulting clonogenic capacity was measured, represented by (**b**) CFU-f number and (**c**) CFU-f diameter. Ag^+^ exposure during initial culture is represented on the x-axis with different Ag^+^ exposures during Stage 2 displayed. Values represent mean (± SEM, n = 3). Statistical analysis by ANOVA; both using appropriate correction for multiple comparisons. Analysis performed for data within each ‘Stage 1’ Ag^+^ concentration, comparing against control, represented by ***p < 0.001, ****p < 0.0001; and against 1 μM Ag^+^ represented by ^$$^*p* < 0.005. Comparison of each ‘Stage 2’ Ag^+^ treatment to its equivalent from the ‘Stage 1’ control group (^#^*p* < 0.05). Effect on CFU-f number when Ag^+^ is applied in MSC-CM. (**d**) Effect of 10 µM Ag^+^ treatment on CFU-f number (± SEM, n = 3) in the presence of non-conditioned and MSC-conditioned medium using primary MSCs (*p* = 0.52) and Y201 MSCs (*p* = 0.81) in the CFU-f assays. Statistical analysis by two-way ANOVA using Sidak’s correction for multiple comparisons, significance indicated by *p < 0.05, **p < 0.001. (**e**) Representative images of crystal violet stained CFU-f for primary MSCs and Y201 MSCs.

Maintenance of MSC clonal expansion capacity following Ag^+^ exposure is necessary for the continued functioning of bone marrow stroma during and subsequent to fracture repair. This property was determined through the secondary seeding of Ag^+^ exposed CFU-f using a two-stage assay design (Fig. 3a). Essentially, CFU-f were exposed to Ag^+^ at 0, 1 and 10 µM concentrations for 14 days (Stage 1). After this time, the surviving CFU-fs from each treatment were trypsinised, re-plated at CFU-f density in fresh plates and re-exposed to Ag^+^ at 0, 1 and 10 µM concentrations for a further 14 days (Stage 2). This protocol enabled us to test the hypothesis that colonies surviving an initial Ag^+^ exposure represented an Ag^+^ resistant MSC subpopulation. Stage 2 CFU-f number for untreated controls was unaffected by previous exposure to Ag^+^. However, the surviving Stage 1 Ag^+^ exposed MSCs remained susceptible to further Ag^+^ exposure in Stage 2 with significant decreases in CFU-f number, irrespective of initial Ag^+^ treatment (Fig. 3b) indicating that Ag^+^ exposure did not select for a resistant MSC subtype.

An inverse correlation was observed between CFU-f diameter and Ag^+^ concentration (Fig. 3c). This relationship was apparent irrespective of pre-exposure to Ag^+^, however the reduction was only significant for those colonies derived from MSCs previously cultured with silver.

### Conditioned medium from MSCs maintains clonogenicity during exposure to Ag^+^

We have found that colony-forming MSCs grown at single cell densities are much more sensitive to Ag^+^ exposure than confluent or near confluent MSC cultures. We hypothesised that this may be due to the decreased availability of secreted, protective signalling factors in CFU-f cultures compared to more densely packed MSC monolayers. To test this hypothesis, conditioned medium (CM) was collected from confluent MSC cultures and applied to the CFU-f assays in the presence and absence of silver ions. We found that the CFU-f number reduction observed at 10 µM Ag^+^ could be rescued by the addition of CM (Fig. 3d). Both human primary MSCs and the Y201 human MSC line (used here as a reproducible model human MSC clonal line^29^) showed recovery of CFU-f number near to that of control when treated with Ag^+^ in CM. There was also a notable increase in colony size of the CM groups compared to unconditioned medium controls (Fig. 3e).

### Glutathione synthesis is upregulated in MSCs of surviving CFU-f in response to Ag^+^

**Figure 4:**
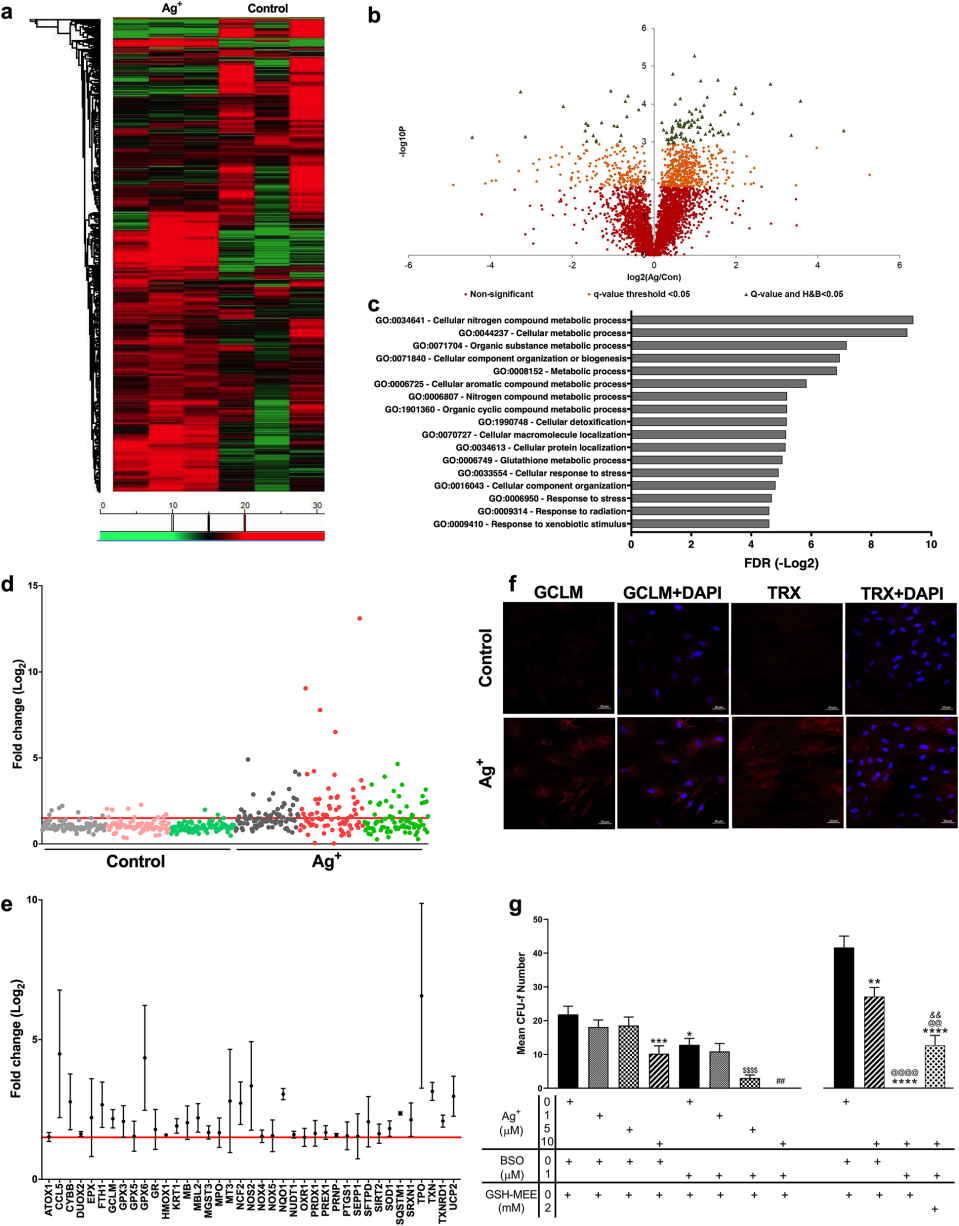
(**a**–**g**) Proteomic and molecular identification of the oxidative stress response of MSCs during exposure to Ag^+^. (**a**) Protein relative abundance heat map from Y201 CFU-f under control and Ag^+^ exposure conditions (green < 15, red > 20). (**b**) Volcano plot of 5050 quantified proteins, negative/positive log_2_ data equate to Ag^+^ group down/upregulated, respectively. 15.19% of proteins were significantly differentially regulated accepting Progenesis QI calculated q-value of < 0.05 (orange), reducing to 2.17% (green) when applying additional multiple test correction to 5% FDR (Benjamini and Hochberg). (**c**) GO terminology of biological processes (FDR < 0.05) associated with differentially upregulated proteins of Ag^+^ cultured Y201 CFU-f. (**d**) Fold change of oxidative stress response genes in 3 primary MSC donors (grey, pink/red and green; dots represent different genes), the red line represents 1.5 fold-change. (**e**) Oxidative stress genes exhibiting ≥ 1.5 fold-change following Ag^+^ exposure versus controls, values are mean ± SEM (n = 3). (**f**) Immunostaining of CFU-f for GCLM and TRX (both red), counterstained with DAPI (blue). Scale bar: 50 µm. (**g**) Ag^+^ induced reduction of CFU-f is potentiated by inhibition of glutathione synthesis with BSO, with recovery aided by exogenous GSH. Results represent the mean CFU-f number ± SEM (n = 3). Statistical analysis by one-way ANOVA using Sidak’s correction for multiple comparisons. Comparisons performed against control represented by *p < 0.05, **p < 0.01, ***p < 0.001, ****p < 0.0001. Additional comparisons made against treatment groups containing the same level of Ag^+^ (e.g. 1 μM Ag^+^ v 1 μM Ag^+^/1 μM BSO), significance indicated compared to 5 μM Ag^+^ ($) and 10 μM Ag^+^ (#). Exogenous GSH in the presence/absence of 10 µM Ag + and 1 µM BSO. Comparisons performed against control as before, with additional comparisons made against Ag^+^ (@) and Ag^+^/BSO (&).

We have shown that a proportion of MSCs persist as CFU-fs following exposure to Ag^+^ ions, suggesting that Ag^+^ may activate survival mechanisms in MSCs, depending on cell density. To identify these mechanisms, Y201 MSCs were used in a proteomic screen of Ag^+^ exposed CFU-f compared to untreated control CFU-f. Liquid chromatography tandem mass spectrometry (LC–MS/MS) proteomic analysis identified 5050 quantifiable proteins, with peak area-based label-free relative quantification applied using Progenesis QI software (Fig. 4a). Of those identified, 89 were significantly upregulated in Ag^+^ exposed CFU-f compared to controls, while 21 were significantly downregulated (FDR < 0.05) (Fig. 4b). Supplementary figures S1(a–d) demonstrate the differentiation of sample groups and consistency of biological replication in the proteomic data set. Gene Ontology (GO) analysis identified several differentially regulated functional responses including several metabolic pathways, as well as cellular detoxification (GO:1990748), response to stress (GO:0006950) and response to xenobiotic stimulus (GO:0009410) (Fig. 4c). Comparison of proteomic data to known KEGG pathways highlighted glutathione metabolism as the major upregulated cellular response to Ag^+^ exposure. Further KEGG pathway analysis drew attention to the upregulation of proteins involved with DNA replication and nucleotide excision repair (PCNA, LIG1, CUL4A).

Validation of the proteomic evidence was provided by quantitative Polymerase Chain Reaction (qPCR) analysis of the oxidative stress response of three primary MSC donors (Fig. 4d). Data were normalised to B2M, which identified 39 genes showing mean fold-change > 1.5 (Fig. 4e). Genes of glutathione synthesis/metabolism (GPX3, GPX5, GPX6, GR, GCLM), peroxiredoxin (PRDX1) and thioredoxin (TXN, TXNRD1) showed activation of these pathways upon Ag^+^ exposure. Additionally, increases in the stress inducible regulator of NRF2, sequestome 1 (SQSTM1), heme oxygenase-1 (HMOX1), NAD(P)H:quinone oxidoreductase-1 (NQO1) and SOD1 were also noted for all donors. GO functional analysis related to biological processes was performed on the qPCR data, with an FDR (false discovery rate) below 5% the threshold for significance (Supplementary Fig. S2). Highlights of those terms include: the removal of superoxide radicals (GO:0019430), response to hydrogen peroxide (GO:0042542), regulation of reactive oxygen species metabolic process (GO:2000377) and glutathione metabolic process (GO:0006749). Furthermore, cellular response to cadmium (GO:0071276) and cellular copper homeostasis (GO:0006878) all indicate at the mechanisms employed to ensure management of metal ions that could result in toxicity. Of those identified, glutathione metabolic process (GO:0006749) was replicated from the proteomic analysis (Supplementary Fig. S3), the only functional process to be so.

Using primary MSCs, we identified increased GCLM immunostaining, a component of glutathione synthesis, and thioredoxin in Ag^+^ exposed CFU-f compared to untreated controls (Fig. 4f). Analysis of whole colony area did not reveal any variation in the expression of either protein.

We used a pharmacological inhibitor of glutathione synthesis, l-buthionine sulfoxamine (BSO), to determine the functional effects of glutathione on CFU-f survival while under Ag^+^ exposure conditions. As observed before, a significant reduction in CFU-f number was noted at 10 µM Ag^+^ in the absence of glutathione inhibition. However with the addition of 1 µM BSO, a significant reduction in CFU-f was observed with Ag^+^  ≥ 5 µM, compared to their equivalent Ag^+^ only control (Fig. 4g). In the case of 10 µM Ag^+^, CFU-f formation was eliminated in the presence of BSO. Recovery of CFU-f number was achieved through the concomitant treatment of 10 µM Ag^+^/BSO with exogenous glutathione (GSH-MEE).

### Effects of silver on adipogenic differentiation is dependent on exposure time

The level of lipid deposition by MSCs as measured by Oil Red O staining revealed a reduction in adipogenesis when exposed to silver compared to controls. While a short term exposure during the initial 3 days of differentiation had no effect, continuous silver treatment during differentiation resulted in significant declines compared to untreated control and short term sliver exposure (*p* < 0.001) (Supplementary Fig. S4a). In contrast, pre-treatment of MSCs with silver during expansion, with subsequent silver-free differentiation, caused a slight but significant increase in adipogenesis compared to control (*p* < 0.05, Supplementary Fig. S4b).

### Osteogenic differentiation of MSCs is unaffected by Ag^+^ exposure

**Figure 5:**
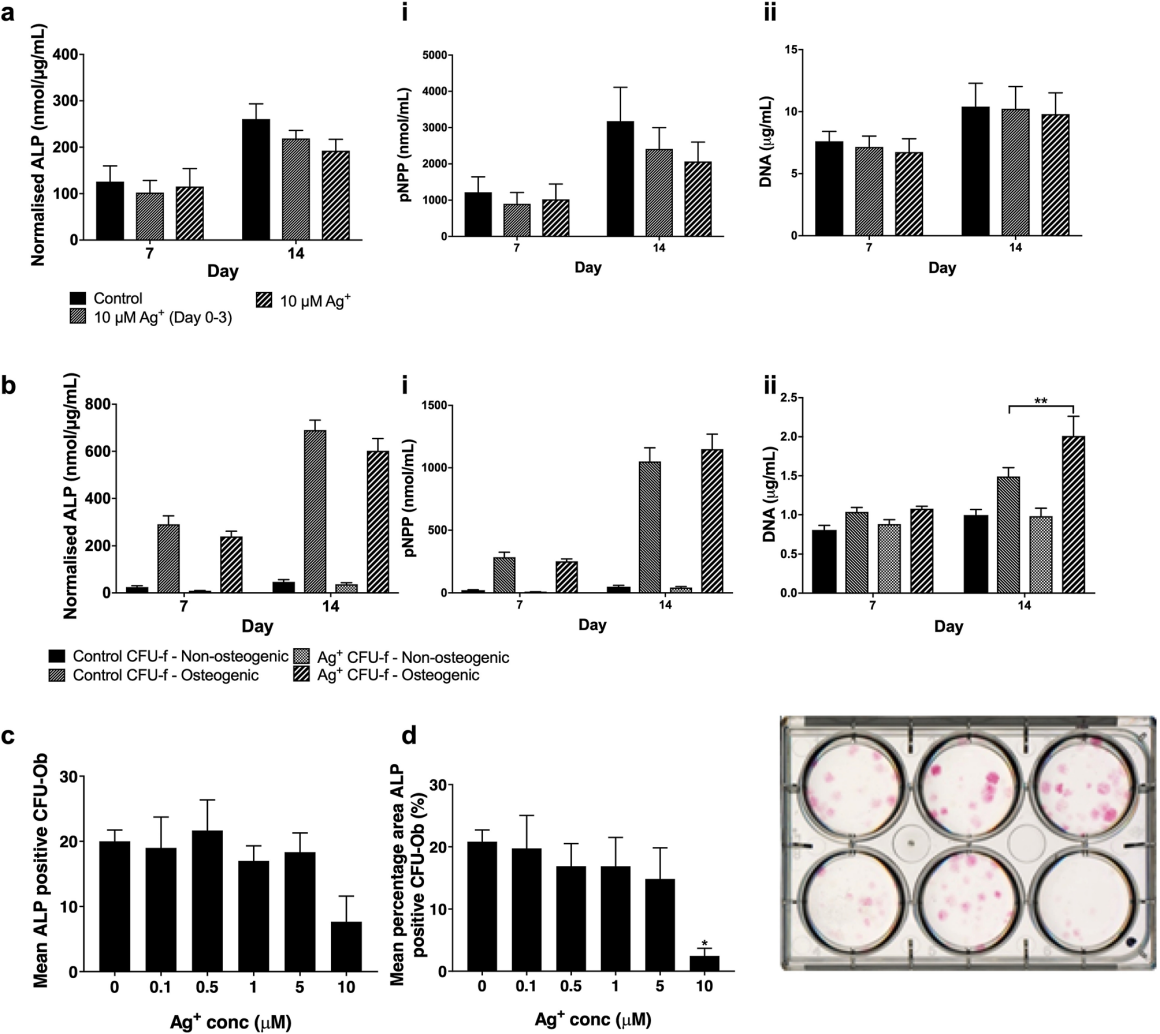
(**a**–**d**) Normalised ALP activity of MSCs following Ag^+^ exposure (**a**) during, and (**b**) prior to differentiation. (**a**) Normalised ALP activity in the presence of 10 μM Ag^+^ for either the initial 3 days of culture (Ag^+^ 10 μM (Day 0–3)) or for the entire differentiation period (Ag^+^ 10 μM). Control cultures received Ag^+^ free osteogenic differentiation medium only. Total ALP activity (i) and DNA quantification (ii) were measured from cell lysates. (**b**) Normalised ALP activity of CFU-f derived MSCs cultured in the presence/absence of 10 μM Ag^+^ prior to osteogenic differentiation in Ag^+^ free medium. Total ALP activity (i) and DNA quantification (ii) were measured from cell lysates. The results represent the mean ± SEM for three donors, performed in triplicate. CFU-Ob, (**c**) number, and (**d**), total percentage area during Ag^+^ exposure. All values are means ± SEM (n = 3). Representative image (right) of CFU-Ob, with Ag^+^ concentrations for plates (L–R): top row, 0, 0.1, 0.5 μM; bottom row, 1, 5, 10 μM. Statistical analysis performed by ANOVA using appropriate correction for multiple comparisons. Significance against control indicated by *p < 0.05, **p < 0.01.

The effect of Ag^+^ exposure on MSC osteogenic differentiation capacity was assessed by measurement of alkaline phosphatase (ALP) activity. Osteogenic differentiation of MSCs was performed in the presence and absence of 10 µM Ag^+^, with ALP activity normalised to DNA determined for short-term (3 day) exposure and continual Ag^+^ exposure groups (Fig. 5a). Cultures treated during the initial 3-day period exhibited a 16% decline from control, while a 26% reduction was observed for cultures treated for the entire differentiation period, however both findings did not reach significance (*p* = 0.582 and *p* = 0.246, respectively) (Fig. 5a and a,i). Furthermore, analysis of DNA data provided additional evidence of the long-term viability of confluent cultures at continual sub-EC_50_ Ag^+^ concentrations (Fig. 5a, ii).

We had previously found that Ag^+^ exposure could inhibit MSC CFU-f capacity. We therefore determined if the osteogenic differentiation of MSCs was affected following CFU-f culture in the presence of Ag^+^. We found that osteogenic capacity (as measured by normalised and total ALP activity) was unaffected by previous exposure to Ag^+^ during CFU-f (Fig. 5b and b,i). However, and in contrast to previous observations, DNA counts (relating to cell number) were significantly elevated in cultures from MSCs previously exposed to Ag^+^ (Fig. 5b,ii).

### CFU-Ob capacity is inhibited during Ag^+^ exposure

MSCs were plated at colony-forming seeding density and then exposed to osteogenic conditions to determine the effect of Ag^+^ exposure on the formation of CFU-Ob. In a manner similar to the effects of Ag^+^ exposure on CFU-f growth, the generation of ALP-positive CFU-Ob was decreased in the presence of continuous Ag^+^. Colony number in the 10 µM Ag^+^ treatment group declined by 62% from control but did not reach statistical significance (*p* = 0.112, Fig. 5c), however total CFU-Ob area was significantly reduced at this sub-EC_50_ concentration (Fig. 5d).

## Discussion

The consequences of infection of orthopaedic trauma devices are both severe for the patient and costly to the healthcare system^2,30^. Although the risk of infection for open tibial fractures is as high as 36%, the primary aim of treating all at risk injuries with implants comprising an anti-microbial technology should not jeopardise successful union^31^. Here, we have investigated the use of silver as a broad spectrum antimicrobial in the prevention of biofilm formation, specifically its elution within an intramedullary setting. We then used the in vivo elution profile data to determine in vitro effects on MSC colony-forming capacity and differentiation, the mechanisms of MSC tolerance to Ag^+^ induced oxidative stress, and the outcomes of fracture healing.

The elution profile of any additive from an implant is of importance for not only efficacy but also determining the safety of such products during pre-clinical evaluations. With regards to testing silver-coated implants, valuable data can be gained from laboratory-based testing, for example using saline or simulated body fluid as carrier solutions. However due to the chemical complexities of using this transition metal, the analysis of silver elution from implants placed in clinically relevant in vivo localities is essential^32,33^. Previous reporting of release profiles from silver technologies have shown a rapid increase in silver at day 2 following sub-cutaneous implantation, declining thereafter from day 6^15^. Consistent with those data, we also found a rapid elevation in silver concentration at the implantation site, additionally correlating this to the concomitant rise in plasma concentrations and a large decline of implant-associated Ag^+^. Importantly, this was mapped during the initial period following implantation and allowed calculation of the maximal Ag^+^ burst release within the free canal space, which was applied in our in vitro investigation. While the elution of some pharmaceuticals has been assessed in this way, to our knowledge, the determination of silver elution from such an implant location has not previously been described^34^. Importantly, the same implants proved capable of reducing in vitro colonisation by *S. aureus* over a 14 day period, a pathogen commonly the cause of implant related infection^3^.

MSCs account for < 0.01% of the cells isolated from the bone marrow, therefore the capacity to proliferate from a low density population provides the niche with a rapid source of progenitors required for skeletal remodelling and repair^35^. The maintenance of a viable population of colony-forming stromal cells is of paramount importance, and the CFU-f assay can be used as an in vitro assessment of this ability^36,37^. Through this assay we revealed a reduction of MSC colony-forming capacity in the presence of sub-toxic Ag^+^ that prompted the analysis of the mechanisms of tolerance employed by the surviving colonies. ROS production following heavy metal ion exposure is regulated by superoxide dismutase (SOD) and catalase (CAT) in addition to several other antioxidant enzymes and their components, such as the predominant source of cellular thiol groups, glutathione^38^. However, variations in oxidative stress protein synthesis between tissues have been identified during in vivo cadmium exposure^39^. In vitro molecular and protein profiling of these cellular mechanisms has ordinarily been performed after short term exposure using confluent cultures, while investigating specific proteins involved in oxidative stress management^40–42^. Significantly, for orthopaedic applications, the data presented here provides an insight into the global mechanisms employed by chronically exposed Ag^+^ tolerant clonogenic MSCs.

The glutathione synthesis inhibitor, BSO, elevates intracellular ROS and sensitises systems to sub-toxic doses of oxidative agents; recovery of cell viability can be achieved through the administration of exogenous glutathione^43,44^. For the first time with MSCs, we used this technique to confirm the findings of the proteomic, bioinformatics and qPCR analysis and in doing so revealed the importance of glutathione to the clonogenic survival of MSCs following exposure to Ag^+^ ions. Our data also highlighted a reduction in colony number during prolonged BSO treatment, although reduction in in vitro cell viability was not observed by other researchers investigating the effects over shorter time periods, with a greater BSO concentration^45,46^. Additionally, the inhibition of the glutathione system potentiated the effect of sub-toxic Ag^+^ concentrations, which overwhelmed the remaining defence mechanisms of the normally sliver-tolerant sub-population.

Oxidative DNA damage resulting from ROS can be induced in cells exposed to Ag^+^^47^. While the risk of transversion mutations is abrogated by several DNA repair mechanisms, the upregulation of proteins involved in global genome nucleotide excision repair were identified in our assessment of MSC clonogenicity following silver treatment. This suggests that neutralisation of ROS alone is not sufficient to enable colony formation in sliver-treated MSCs and that other pathways are employed in order to maintain the MSC population.

Increased oxidative stress impairs fracture healing in rodents^48,49^. In a study by Lippross et al. a decline in the quality of bone remodelling was described and has been supported by evidence of reduced osteogenic differentiation of MSCs in vitro while under conditions of oxidative stress, including those caused by Ag^+^ exposure^49–52^. Osteoblast activity plays an important role in the formation of the hard callus during secondary fracture healing, an event that occurs several weeks following the initial injury. We determined that the retention time of silver within the bone was minimal in our model, additionally, we found that adipogenesis was differentially altered dependent on the timing of exposure. While a limitation of our study is our measurement of one indicator of osteogenesis (ALP), with further work investigating other markers, for example by qPCR, required to provide more robust information, we report that little effect on the osteogenic capacity of confluent MSCs and cell viability was observed even while under the 10 µM Ag^+^ conditions, a concentration that is in line with the maximum silver elution characteristics measured using the rat in vivo model. Of interest, however, was the significant decline in MSC clonogenicity that was discovered while under these same conditions. The importance of MSC number at a fracture site was highlighted by Hernigou et al. and it is conceivable that our observation of reduced clonogenicity during conditions of oxidative stress may explain the reports of reduced healing in fracture models with localised induced ROS^48,49,53^. Further investigation of MSC function following in vivo exposure to the silver coated implants would have provided valuable additional insight.

Together, our data indicate that clinically relevant silver concentrations remain sub-toxic to MSCs at high density, but can result in a decreased colony-forming capacity. Tolerant CFU-fs show upregulated mechanisms to neutralise ROS and minimise the impact of DNA damage, with the implication that those cells maintain their osteogenic capacity, allowing the progression of fracture healing. These findings indicate that silver may have a role to play in reducing the incidence of fracture fixation related infection, bringing potential benefits to both patients and healthcare system.

## Materials and methods

The following methods describe the investigation of Ag^+^ release from intramedullary implants, translating this to in vitro methods that assessed the effect on MSC function.

### Preparation and characterisation of silver coated implants

Titanium alloy (Ti-6Al-4V) rods (20 mm length × 1.1 mm diameter) were passivated in 10 M sodium hydroxide at a temperature of 60 °C (pins, 1 h 50 min) followed by incubation in 0.1 M silver nitrate (Alfa Aesar) at 60 °C for 1 h, to achieve a targeted silver dose of ~ 50 µg/cm^2^. All implants were gamma sterilised prior to surgery (25–40 kGy).

Total silver analysis was determined pre- and post-implantation (where appropriate). Individual implants were placed in 1:2 (v/v) nitric acid/dH_2_O and incubated overnight at room temperature (RT). Solutions were vortexed and diluted 1:1000 (v/v) in 1% nitric acid. Silver content was determined against an Ag^+^ standard (0.2–20 ppb) using the Agilent 8800 ICP-MS Triple Quad (Stockport, UK) in the presence of a 500 ppb rhodamine internal standard.

### Extended time-point repeat challenge microbiology testing of silver coated pins

Silver coated pins in cryovial tubes (NUNC, UK) were inoculated with 1 mL *Staphylococcus aureus* ATCC 25923 (1 × 10^4^ CFU/mL) in 10% Foetal Bovine Serum (FBS)/PBS and incubated horizontally (37 °C with agitation at 150 rpm). Each time point (24 h, 7, 14, 21 and 33 days, n = 5/time point) received a 50% media change at 3–4 day intervals, replacing 500 μL of test suspension with and equivalent volume of fresh 10% FBS in PBS (Note: the 24 h time point received no media change and 33 day sample received no media change between days 18 and 27).

For each time point, samples allotted for analysis were transferred to a fresh cryovial and 1 mL of fresh inoculum added to each. Following a further 24 h incubation, the pins were placed in to clean tubes, washed 6 × with sterile PBS before transfer to neutraliser (0.85% NaCl, 0.4% sodium thioglycollate, 1% Tween 20) and the colonising bacteria from the surface were recovered as described in ASTM E2871-13. Briefly this comprised vortex mixing for 30 s, followed by sonication for 30 s (45 kHz), this was repeated, with a final vortex before serial dilution in MRD and plating on to Petrifilm (3M, UK). All Petrifilm were incubated for at least 48 h at 32 °C before counting. Twenty-four hour samples underwent the same recovery process, without the additional 24 h re-challenge following initial incubation.

### In vitro cell culture

Mesenchymal stem/stromal cells (MSCs) of human origin were isolated from bone marrow aspirate (Lonza) or excised bone donated by patients undergoing primary arthroplasty surgery (NHS, Clifton Park Hospital, UK). Informed consent was obtained from all subjects. Collections and all methods were carried out in accordance with relevant guidelines and regulations under approval from the University of York Biology Ethical Committee Review Board and NHS Local Research Ethics Committee. Clonal MSCs, termed Y201, were generated from primary bone marrow derived MSCs engineered to overexpress human telomerase reverse transcriptase (hTERT)^29^. The use of Y201 MSCs as a model system has been indicated where appropriate. Both primary MSCs and Y201 MSCs were expanded in Dulbecco’s modified Eagle’s medium (DMEM) supplemented with 10% FBS, 100 Units/mL Penicillin and 100 μg/mL Streptomycin (37 °C, 5% CO_2_). Human osteoblasts (hObs) were purchased from PromoCell (Germany) and cultured in osteoblast growth media (PromoCell). Cells were passaged using 0.25% Trypsin–EDTA once 90% confluent. All reagents purchased from Sigma (Poole, UK) unless otherwise stated.

### In vitro viability and proliferation

Primary MSCs seeded at 3.125 × 10^4^ cells/cm^2^ in 96-well plates were incubated for 24 h before addition of an Ag^+^ dose response (range 8.75–100 μM, Ag_2_SO_4_, Alfa Aesar, Heysham, UK). After a further 24 h, WST-1 reagent was added and the optical density read at 440 nm and 600 nm after 1 h. Results were converted to viability as a percentage of the untreated control. Proliferation over 72 h was assessed using 5-ethynyl-2′-deoxyuridine (EdU) incorporation (ThermoFisher, Loughborough, UK). MSCs were seeded for 24 h in to 24-well plates (2.5 × 10^3^ cells/cm^2^) before addition of Ag^+^ (0–10 µM). After 72 h, cultures were counterstained with Hoechst and image analysis performed using ImageJ, providing the percentage of EdU positive cells.

### CFU-f and CFU-Ob formation

Primary MSC cultures at clonal density (10 cells/cm^2^) were established in DMEM + 20% HyClone FBS (GE Healthcare, Little Chalfont, UK) to generate CFU-f (colony-forming unit fibroblast). As for the assessment of viability and proliferation, medium was changed 24 h after seeding for that containing Ag^+^ (0–10 µM). Medium was changed twice per week, with one subset of plates receiving Ag^+^ for the first 3 days only, after which medium was without silver. Colony formation (defined as ≥ 50 cells) was assessed after 14 days following crystal violet staining (0.05% w/v crystal violet) for 20 min.

CFU-Ob (colony-forming unit osteoblast) were generated as described for CFU-f, but supplementing the medium with osteogenic additives at the medium change 24 h after seeding (50 μg/mL l-ascorbic acid, 10^–8^ M dexamethasone, 3 mM β-glycerophosphate). Alkaline phosphatase (ALP) positive colonies were identified after 14 days using 1% Fast Red TR/0.2% Naphthol AS-MX applied for 2 min.

### Differentiation of MSCs

Primary MSCs were culture expanded, seeded in to well-plates for 24 h prior to the addition of osteogenic differentiation medium (described above) ± 10 µM Ag^+^. As before, one group received Ag^+^ free medium after 3 days while maintaining 10 µM Ag^+^ in the second treatment group. Adipogenic differentiation media (10^-6^ M Dexamethasone, 500 µM 3-Isobutyl-1-methylxanthine, 1 µg/mL Insulin from bovine pancreas, 100 µM Indomethacin) and osteogenic media, were changed twice per week with time points of 21 days (adipogenic) and seven and 14 days (osteogenic). Controls underwent the same medium changes (with/without Ag^+^) and time points.

Adipogenic cultures were rinsed (1xPBS) and fixed for ten minutes in 10% formaldehyde (Polysciences, USA) followed by a rinse with dH_2_O and the addition of Oil Red O working solution for five minutes. The stain was removed and the wells washed with 60% Isopropanol before a final rinse with tap water. Lipid bound Oil Red O was quantified through removal with 99% Isopropanol and the optical density measured at 520 nm (Multiskan GO, Thermo Fisher, UK,). For osteogenic analysis, at the specified time points, cultures were lysed and ALP activity analysed against a p-nitrophenol standard (pNP, 0–250 nmol/mL) using a pNPP substrate (5 mM). Optical density (405 nm) was measured after 1 h (37 °C). Data were normalised to DNA, quantified using PicoGreen (ThermoFisher), measured at Ex: 485 nm/Em: 538 nm. Differentiation following MSC expansion as CFU-f was determined, with MSCs seeded at clonal density ± 10 μM Ag^+^ for 14 days, before differentiation in Ag^+^ free medium. Time points and analysis were as previously described.

### CFU-f formation following Ag^+^ exposure

The continued clonogenic capacity of MSCs following Ag^+^ treatment and the existence of an Ag^+^ tolerant subpopulation that maintained colony formation under further Ag^+^ exposure were assessed using a two-stage CFU-f assay (see Fig. 3a for schematic description). In brief, primary MSCs seeded at clonal density were cultured in medium (0, 1 or 10 µM Ag^+^) and assigned as ‘Stage 1’. Medium was changed twice per week for 14 days, after which, cells from the same treatment group (e.g. control) were pooled. MSCs were re-seeded in to further 6-well plates at clonal density (‘Stage 2’) and medium (± Ag^+^) changed as before. After 14 days, the colonies of the ‘Stage 2’ plates were counted and diameters measured. Assessment of CFU-f diameter was performed using the Zeiss Zen 2.3 Lite software.

### CFU-f formation in conditioned medium

The effect of environmental signals on MSC (primary and Y201 MSCs) clonogenicity was determined by culturing clonally seeded MSCs in conditioned medium (MSC-CM). Tissue culture flasks of MSCs at 70% confluence were rinsed with phosphate buffered saline (PBS) and further culture in serum free DMEM for 24 h. Medium was removed, centrifuged and filtered (0.45 µm) and HyClone FBS added to give 20% (stored 2–8 °C). ‘Non-conditioned’ (i.e. basal) or ‘Conditioned’ medium were used during media changes (± 10 µM Ag^+^) with colonies assessed as before after 14 days.

### qPCR of oxidative stress pathway

RNA was extracted from primary MSC-generated CFU-f (± 10 µM Ag^+^). CFU-f were pelleted and RNA isolated according to RNeasy Mini Kit protocol (Qiagen, Manchester, UK). cDNA synthesis was performed with 500 ng mRNA per sample reaction using RT^2^ First Strand Kit (Qiagen). QPCR was performed using the RT^2^ Human Oxidative Stress PCR Array (Qiagen, Cat: PAHS-065Z) with SYBR Green detection run on the Thermo Fisher Quantstudio 3 Real-Time PCR System (95 °C for 10 min followed by 40 cycles of 95 °C for 15 s, 60 °C for one minute, a final melt curve of 95 °C for 15 s, 60 °C for one minute, 95 °C for one second). Fold-changes were calculated for each donor, normalising to β-2-microglobulin (B2M).

### Global proteomic analysis of CFU-f

Ag^+^ cultured Y201 CFU-f were washed and lysed in urea lysis buffer added at 5:1 (v:v) of the cell pellet before undergoing three bursts of microtip sonication at 15 watts, cooling on ice for one minute between bursts. Reduction, alkylation, trypsin digestion and peptide fractionation was performed as described by Geoghegan et al*.* for *Aedes aegypti* midguts^54^. Peptide fractions were loaded onto an UltiMate 3000 RSLCnano HPLC system (Thermo) equipped with a PepMap 100 Å C_18_, 5 µm trap column (300 µm × 5 mm Thermo) and a PepMap, 2 µm, 100 Å, C_18_ EasyNano nanocapillary column (75 μm × 500 mm, Thermo). Flow rate was 300 nL/min and the column temperature 40 °C. Separation used gradient elution of aqueous 1% (v:v) formic acid (solvent A) and aqueous 80% (v:v) acetonitrile containing 1% (v:v) formic acid (solvent B). The linear multi-step gradient profile was: 3–10% B over 7 min, 10–35% B over 30 min, 35–99% B over 5 min and then proceeded to wash with 99% solvent B for 4 min. The nanoLC system was interfaced with an Orbitrap Fusion hybrid mass spectrometer and positive ESI–MS and MS^2^ spectra were acquired using Xcalibur software (version 4.0, Thermo). MS^1^ spectra were acquired in the Orbitrap with Easy-IC: 120,000 resolution, scan range: *m/z* 375–1500. Data-dependent acquisition was performed in top speed mode using a 1 s cycle and 50 s dynamic exclusion. MS^2^ spectra were acquired in the linear ion trap with HCD activation energy of 32%. Peak area derived relative quantification was obtained from non-conflicting precursor intensities using Progensis QI as detailed previously^55^. Relative abundances were normalised between high pH C_18_ fractions using total ion intensity. Database searching was performed against the human subset of the UniProt database (Date 20180123; 20,244 sequences; 11,338,534 residues) using Mascot (Matrix Science Ltd., version 2.5.1), filtering identifications through the Percolator algorithm to achieve a global 1% FDR. Accepted quantifiable proteins were further filtered to require a minimum of two unique peptides. Multiple test corrected q-values for differential abundance were calculated in Progenesis QI with the Hochberg and Benjamini FDR estimation additionally applied for further stringency.

### Immunocytochemistry of CFU-f (GCLM and TRX)

Detection of γ-glutamylcysteine synthetase (GCLM) and thioredoxin (TRX) was in CFU-f of primary MSCs cultured with Ag^+^ on glass coverslips (using a HeLa cell positive control). Slides were fixed with ice cold 100% methanol (10 min) rinsed with PBS and incubated with 5% goat serum/PBS at room temperature (1 h). Primary antibody incubation (1 h, RT) also occurred in 5% goat serum/PBS 10 μg/mL polyclonal rabbit anti-GCLM IgG (Cat: NBP1-33405, RRID: AB_2107841, Novusbio, UK) and 4 μg/mL polyclonal rabbit anti-thioredoxin IgG (Cat: NBP2-48873, RRID AB_2819005, Novusbio). Slides were rinsed before incubation with 10 μg/mL Alex fluor 647-conjugated goat anti-rabbit IgG (H + L) cross adsorbed (1 h, RT; Cat: A-21244, RRID: AB_2535812, ThermoFisher) and counterstained with 2 μg/mL DAPI. Coverslips were mounted in aqueous mounting media (Abcam, Cambridge, UK). All immunofluorescence imaging was performed using the Zeiss Axio Imager.M2 LSM 710 confocal microscope (Carl Zeiss, Cambridge, UK).

### Inhibition of glutathione synthesis and addition of GSH-MEE

CFU-f from primary MSCs were cultured with Ag^+^ (0, 1, 5 and 10 μM) in the presence/absence of a pharmacological inhibitor of γ-glutamylcysteine synthetase, l-buthionine-sulfoxamine (BSO, 0.01, 0.1 1 μM). Recovery the glutathione pathway in BSO treated cultures was through media supplementation with 2 mM GSH-MEE. Media were changed twice per week with CFU-f number assessed at 14 days.

### In vivo Ag^+^ release from intramedullary implants

Study design was approved by the Smith and Nephew Animal Welfare and Ethical Review Board in compliance with the Animals (Scientific Procedures) Act 1986, taking into consideration the requirements for reduction, replacement and refinement. Twelve male Sprague Dawley rats (250–300 g) were randomised in to four groups consisting of three animals per group. Each group was allocated to a time point of 24, 48, 72 h or 28 days. Animals were group housed in temperature controlled rooms at 21 ± 2 °C with a relative humidity of 55 ± 10% and artificial lighting cycle of 12 h light/dark. Sub-cutaneous antibiotic prophylaxis (Septrin, 24 mg/kg), was provided prior to surgery and twice per day for the initial 2 days of the study. In addition, intra-peritoneal injections of Buprenorphine analgesia (0.05 mg/kg) and medetomidine sedative (0.1 mg/kg) were provided. Following surgery, buprenorphine (0.05 mg/kg) was administered every eight hours for a minimum of 48 h. Animals were anaesthetised using an Isoflurane/Oxygen/Nitrous oxide mixture. Access to the femoral canal was via an entry hole in the inter-condylar notch of the left hind leg. The test article was inserted and the wound closed using absorbable sub-articular sutures. Following surgery, the animals underwent X-rays and were returned to individual housing to recover, after which they were returned to the group. Animals were sacrificed at allocated time points and plasma samples prepared from whole blood collected in heparin sodium salt anticoagulant. The femurs were removed and the implants retrieved.

### Silver quantification of plasma and femurs

Plasma samples were diluted 1:25 (v/v) in 1% nitric acid and the Ag^+^ content determined against a matrix matched standard [0.05–2 ppb, 4% rat plasma (Innovative Research)]. Femurs were heated to 700 °C for four hours and mixed with a 1:2 (v/v) nitric acid/dH_2_O solution and left overnight at RT. Samples were diluted 1:200 (v/v) in 1% nitric acid and Ag^+^ quantified as before (0.2–20 ppb).

### Statistical analysis

Data were expressed as the mean ± SEM. In vitro experiments were performed with a minimum of three primary donors. Data were interrogated as stated in the individual methods using appropriate multiple comparison test. Statistical analysis was performed using GraphPad Prism 7 software (GraphPad Software, La Jolla, CA, USA).

## Supplementary information

Supplementary Information.
