# Supplementary material for: Identification of mesenchymal stromal cell survival responses to antimicrobial silver ion concentrations released from orthopaedic implants
Source: Sci Rep. 2020 Nov 3;10:18950. doi: 10.1038/s41598-020-76087-1 (PMC7609692; doi:10.1038/s41598-020-76087-1)
Supplement: Supplementary file 1 — Supplementary Information. [file 41598_2020_76087_MOESM1_ESM.docx]

**Identification of mesenchymal stromal cell survival responses to antimicrobial silver ion concentrations released from orthopaedic implants**

Paul Souter^1^, John Vaughan^1^, Kerry Butcher^1^, Adam Dowle^2^, Jim Cunningham^1^, James Dodd^1^, Michael Hall^1^, Darren Wilson^1^, Alan Horner^1^, Paul Genever^2^*

* Corresponding author: paul.genever@york.ac.uk

^1^ Smith & Nephew plc, 101 Hessle Road, Hull, HU3 4DJ, UK

^2^ Department of Biology, University of York, Wentworth Way, York, YO10 5DD, UK

**Supplementary data**

Table S1: **Calculation of plasma volume, total Ag+ and maximum Ag+ concentration within the intramedullary canal for study animals from Day 1**. Maximum Ag^+^ concentration (μM) within the free canal space was calculated for each animal using a canal volume measurement of 62.4 μL and an implant volume of 19 μL (molecular weight of silver = 107.9). Total plasma Ag^+^ (μg) determined using volume constant 4.1 mL/100g,^1^ allowing total accounted Ag^+^ measurement (μg) for each animal at Day 1.

|  | Replicates | | |  |  |
| --- | --- | --- | --- | --- | --- |
|  | 1 | 2 | 3 | Mean | SEM |
| Animal weight (g) | 289 | 290 | 332 |  |  |
| Total plasma volume (mL) | 11.85 | 11.89 | 13.61 |  |  |
| Ag^+^ remaining on implant (μg) | 13 | 8 | 11 | 10.7 | 1.45 |
| Ag^+^ in plasma (μg/mL) | 0.0154 | 0.0266 | 0.0151 | 0.019 | 0.004 |
| Total Ag^+^ from femur (μg) | 8.00 | 2.00 | 8.00 | 6.00 | 2.00 |
| Ag^+^ eluted from implant (μg) | 22.8 | 27.8 | 24.8 | 25.16 | 1.45 |
| Ag^+^ concentration within free canal volume (μM) | 4.92 | 6.00 | 5.35 | 5.42 | 0.31 |
| Total plasma Ag^+^ (μg) | 0.182 | 0.316 | 0.206 | 0.235 | 0.072 |
| Total Ag^+^ accounted for (μg) | 21.18 | 10.32 | 19.21 | 16.90 | 3.34 |
| Total Ag^+^ unaccounted for (μg) | 14.65 | 25.51 | 16.62 | 18.93 | 3.34 |


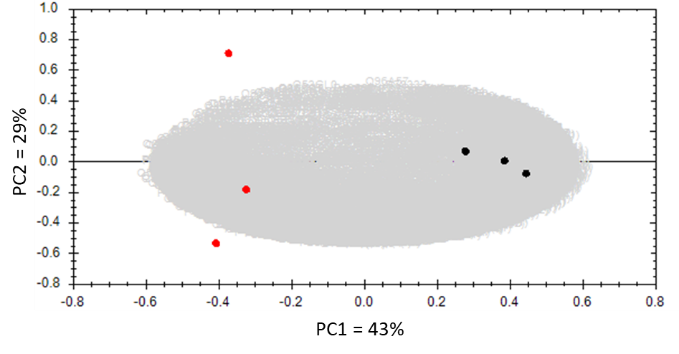

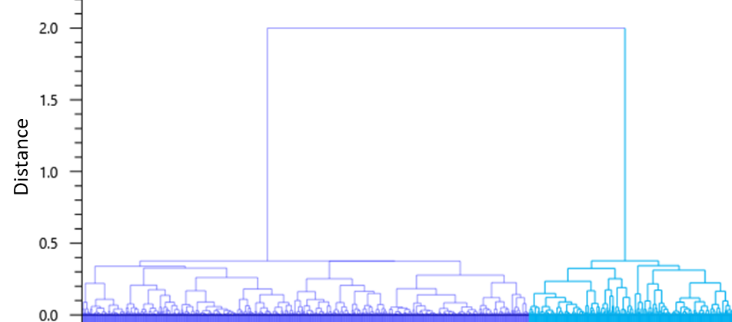

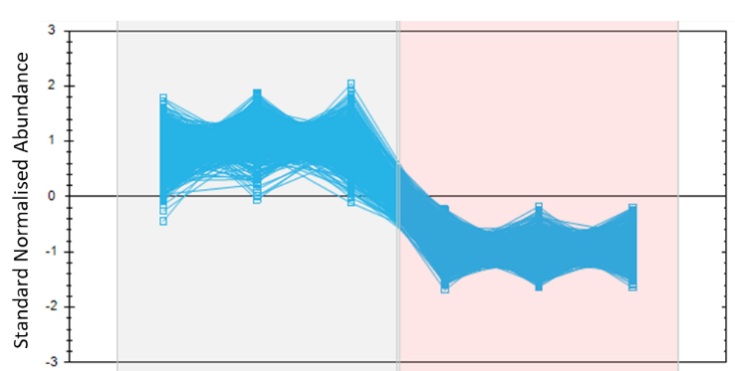

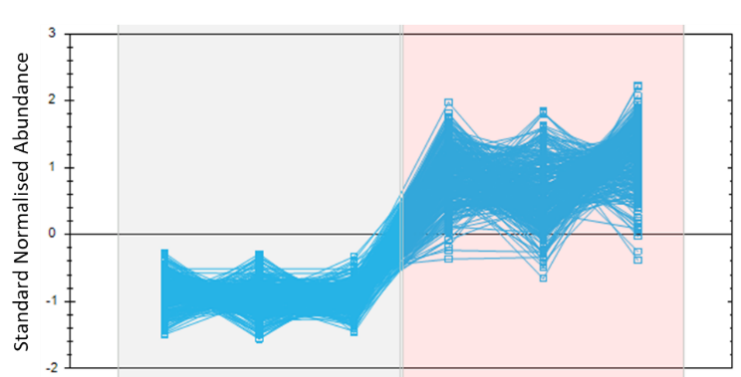


**a**

**b**

**c**

**d**

Figure S1a-d: **Visualisation of proteomic differences between Ag^+^ exposed and control CFU-f samples**. (**a**) Principle component analysis showing separation of Ag^+^ exposed (black) and control samples (red) in label-free quantitative proteomic data. Grey shading shows protein accessions represents relative loading to principle components from each protein identity. (**b**) Dendrogram showing separation of protein clusters on the basis of relative protein abundance in Ag^+^ exposed and control samples, plotting only proteins called as significantly different (q<0.05). Deep blue lines indicate proteins of greater abundance on Ag^+^ treatment and light blue lines are those with lower abundance after treatment. Standardised normalised protein abundance plots for the clusters called as higher (**c**) or lower (**d**) abundance on Ag^+^ exposure are presented showing the consistency of change between the three biological replicates; points on left hand side (grey box) represent protein abundance measurements in Ag^+^ treated samples and those in the pink shaded right hand side are from control samples.


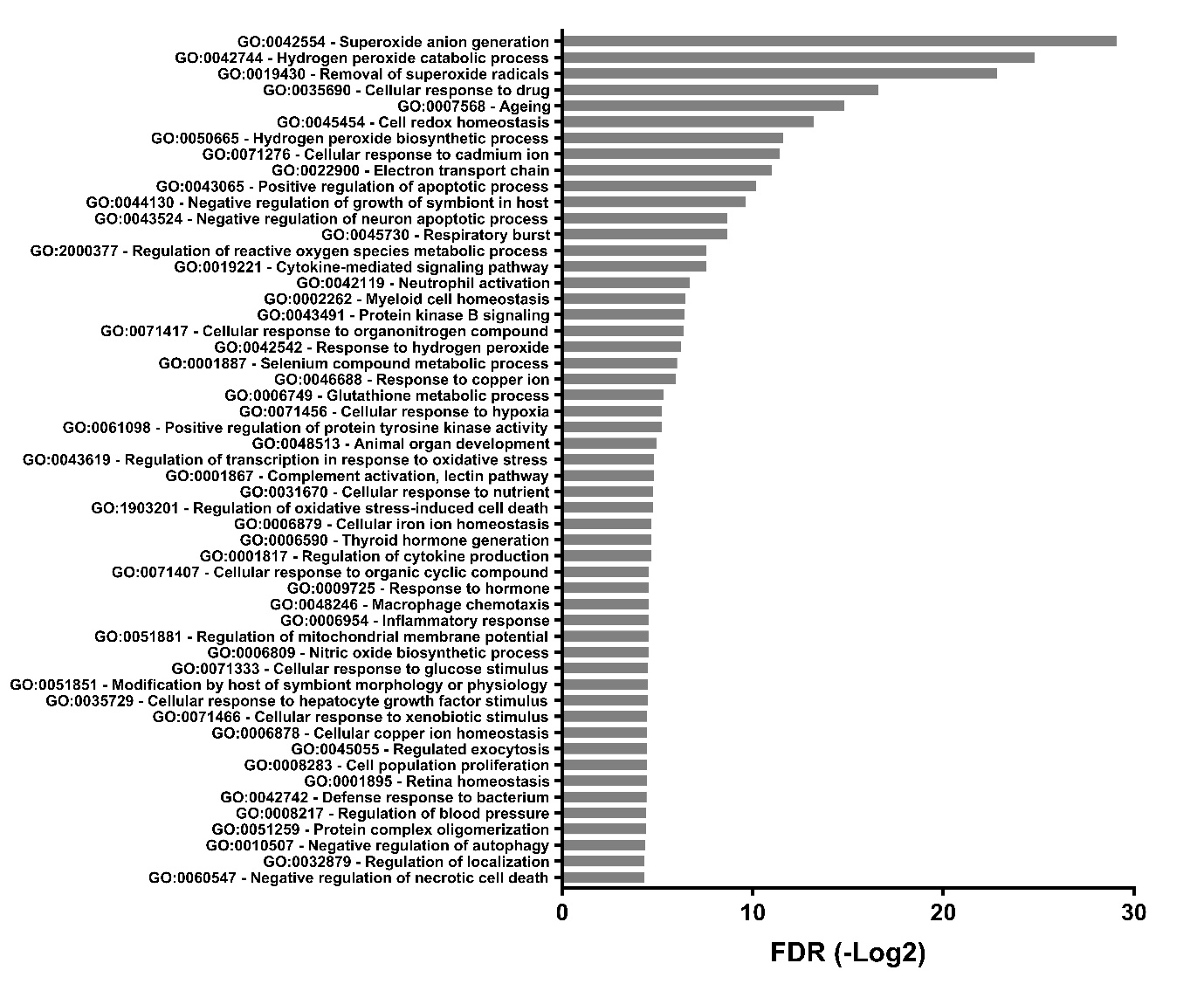


Figure S2: **GO term analysis of differentially upregulated proteins from Ag^+^ exposed Y201 MSC generated CFU-f compared to untreated controls**.


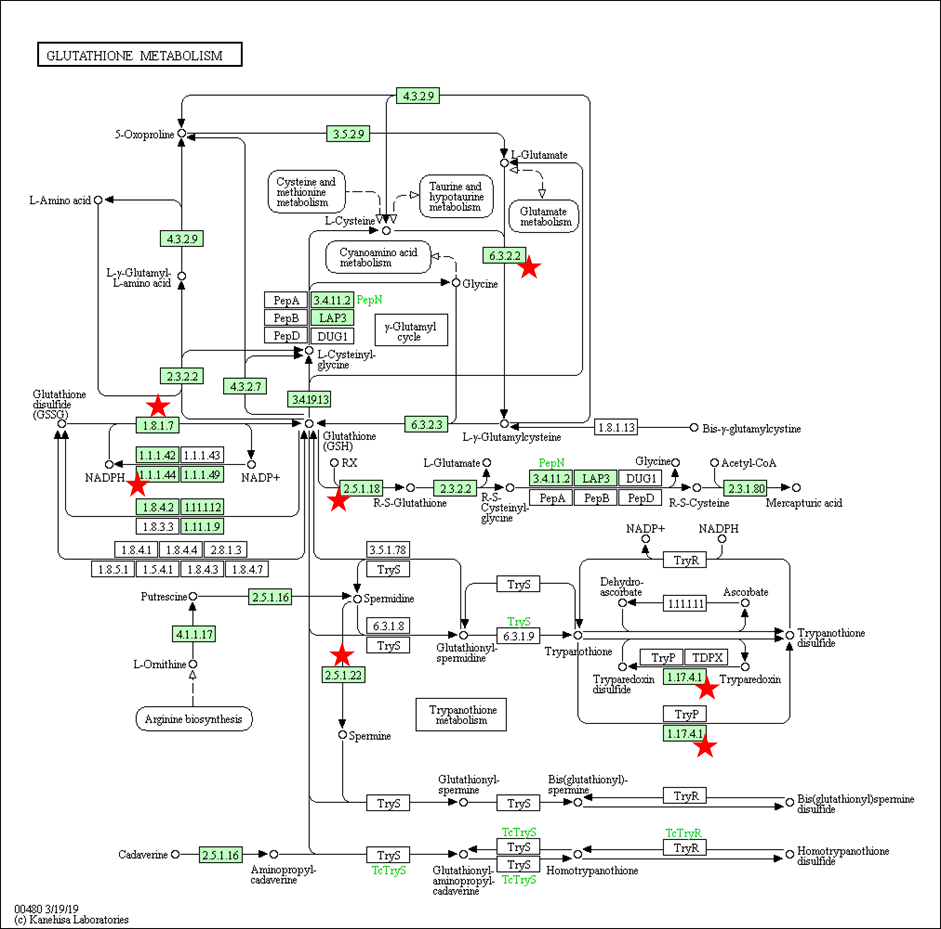


Figure S3: **Proteins quantified as greater abundance in Ag^+^ treated MSCs marked with red stars and mapped onto the KEGG pathway for glutathione metabolism using DAVID bioinformatic resources version 6.8**. The Benjamini-corrected p-value for glutathione metabolism enrichment in proteins called as more abundant on Ag^+^ treatment was 1.9x10^-3^ ^2–4^.


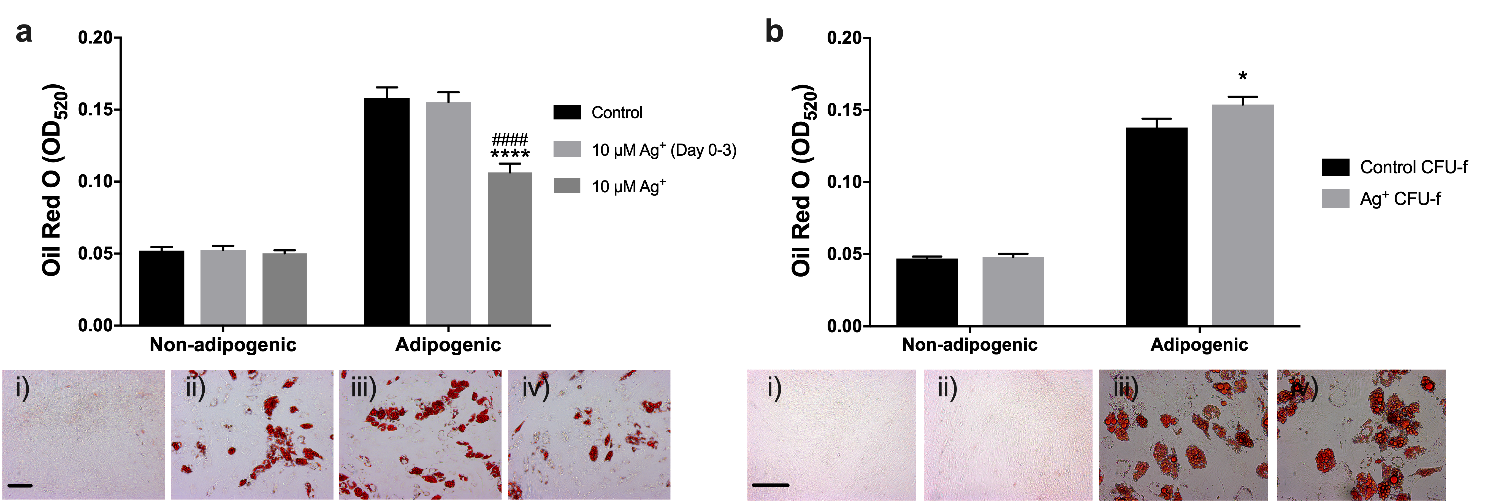


Figure S4a-b: Adipogenic differentiation following Ag^+^ exposure (**a**) during, and (**b**) prior to differentiation as determined by optical density quantification of lipid deposits stained using Oil Red O. (**a**) Adipogenic differentiation in the presence of 10 μM Ag^+^ for either the initial three days of culture (Ag^+^ 10 μM (Day 0-3)) or for the entire differentiation period (Ag^+^ 10 μM). Control cultures received Ag^+^ free adipogenic differentiation medium only. Results represent the mean Oil Rd O OD_520_ ±SEM for four donors performed in triplicate. Representative images of Oil Red O stained cultures are provided as follows: i) non‑adipogenic, 10 μM Ag^+^; ii) adipogenic, control; iii) adipogenic, 10 μM Ag+ (Day 0-3); iv) adipogenic, 10 μM Ag^+^; (scale bar = 100 μm). (**b**) Differentiation of CFU-f derived MSCs cultured in the presence/absence of 10μM Ag^+^ prior to adipogenic differentiation in Ag^+^ free medium. The results represent the mean OD_520_ ±SEM for three donors, performed in triplicate. Representative images of Oil Red O stained cultures (HS7674) are provided as follows: i) non‑adipogenic, control CFU-f; ii) non-adipogenic, Ag^+^ CFU-f; iii) adipogenic, control CFU-f; iv) adipogenic, Ag^+^ CFU-f; (scale bar: non‑adipogenic = 250 μm, adipogenic = 50  μm). Statistical analysis performed between all groups for both non‑adipogenic and adipogenic by two‑way ANOVA using appropriate correction for multiple comparisons. Significance against control represented by * *p*<0.05, **** *p*<0.0001, significance against 10 μM Ag^+^ (Day 0-3) represented by #### = *p*<0.0001.

References

1. Probst, R. J. *et al.* Gender differences in the blood volume of conscious Sprague-Dawley rats. *J. Am. Assoc. Lab. Anim. Sci.* **45**, 49–52 (2006).

2. Kanehisa, M. & Goto, S. KEGG: Kyoto Encyclopedia of Genes and Genomes. *Nucleic Acids Res.* **28**, 27–30 (2000).

3. Kanehisa, M., Sato, Y., Furumichi, M., Morishima, K. & Tanabe, M. New approach for understanding genome variations in KEGG. *Nucleic Acids Res.* **47**, D590–D595 (2018).

4. Kanehisa, M. Toward understanding the origin and evolution of cellular organisms. *Protein Sci.* **28**, 1947–1951 (2019).
